# *ERECTA* Regulates Cell Elongation by Activating Auxin Biosynthesis in *Arabidopsis thaliana*

**DOI:** 10.3389/fpls.2017.01688

**Published:** 2017-09-27

**Authors:** Xiaoya Qu, Zhong Zhao, Zhaoxia Tian

**Affiliations:** School of Life Sciences, University of Science and Technology of China, Hefei, China

**Keywords:** *Arabidopsis*, *ERECTA*, auxin, hypocotyl length, cell elongation

## Abstract

The *ERECTA* family genes, *ERECTA (ER), ERECTA-LIKE1 (ERL1)*, and *ERECTA-LIKE2 (ERL2)*, encode leucine-rich repeat receptor-like kinases in *Arabidopsis thaliana.* Knocking out these three genes can cause severe phenotypes, which indicates that they play significant roles in plant growth and development. However, the molecular mechanism within remains unclear. Here we show that the short hypocotyl phenotypes of *er erl1 erl2* mutants are mainly due to the defects of cell elongation rather than the cell division. In contrast, in the *ERECTA* overexpression transgenic plants, the hypocotyl length is increased with elongated cells. Moreover, we show that the *er erl1 erl2* triple mutant contains a low level of auxin, and the expression levels of the key auxin biosynthesis genes are significantly reduced. Consistent with this observation, increasing exogenous or endogenous auxin levels could partially rescue the cell elongation defects of the *er erl1 erl2* triple mutant. Therefore, our results provide a molecular basis for auxin mediated *ERECTA* control of the hypocotyl length in *Arabidopsis thaliana*.

## Introduction

Plant cell size is one of the most important features in plant morphology, which is controlled strictly by the inheritance and influenced by the external environment. The cell size directly correlated with cell division and cell elongation. Schleiden and Schwann (1838-1839) established the “cell theory,” which regard that the cell division and cell elongation activities determine the growth and development of living organism. Cell elongation is very important for the process of plant differentiation and morphogenesis of plant organs. The growth of hypocotyls in *Arabidopsis* is the elongation and division of hypocotyl cells in essence. In particular, the cell elongation growth plays a decisive role in hypocotyl length (Stuart et al., 1977; Fenwick et al., 1999). The hypocotyl is an important structure connecting root, shoot tip and leaves, and also an important channel for transporting water, nutrients and signaling molecules in *Arabidopsis*. At the same time, hypocotyls are very sensitive to endogenous signal molecules and external environment signal (Gray et al., 1998; Nozue et al., 2007; de Lucas et al., 2008; Christie et al., 2011; Sun et al., 2012). The morphological structure of *Arabidopsis thaliana* hypocotyl is simple, consisting of more than 20 cells in longitudinal direction, which is below cotyledons to the above of radicle. Because most of these cells are formed in the embryonic stage, only a few are produced by cell division after germination, suggests that the hypocotyl growth is mainly caused by the cell elongation (Gendreau et al., 1997). Therefore, the hypocotyl of *Arabidopsis* has become an important model system for studying plant cell elongation due to its simple structure and important physiological functions.

Plant hormones are important regulatory signal molecules of hypocotyl elongation, and auxin is one of the most important types. For a single cell, the main physiological function of auxin is to regulate cell elongation, and the effect of auxin on acceleration of cell elongation is very rapid, and the lag time from treatment to effectiveness is only about 10 min. Previous studies have found that mutants with excessive synthesis of auxin in light exhibit a longer hypocotyl length, while mutants with deficient synthesis of auxin have shorter hypocotyl length (Romano et al., 1991; Jensen et al., 1998; Zhao et al., 2001; Cheng et al., 2006). This indicates that an appropriate concentration of auxin is essential for the growth and development of hypocotyls. It has now been shown that the effect of auxin on hypocotyl length is achieved by stimulating the degradation of IAA3, which in turn causes the ARF transcription factor to be released (Vernoux et al., 2011; Oh et al., 2014). ARF transcription factors, especially ARF6 and ARF8, combine the signals from endogenous development and exogenous environments to control the elongation of hypocotyl cells by activating the expression of cell elongation factors such as *PRE*, *SAUR* family genes (Bai et al., 2012; Oh et al., 2014; Challa et al., 2016).

The intercellular communication is very important for the formation of normal tissues, organs and organisms of plants. The cell surface receptor protein kinase located on the cytoplasmic membrane plays a vital role in the initiation of cell signal transmission. The *ERECTA* (*ER*) gene in *Arabidopsis* encodes a leucine-rich repeat receptor-like protein kinase, whose protein structure consists of a hydrophobic signal peptide in the extracellular domain, a leucine repeat sequence for protein interaction, a single transmembrane domain, and intracellular serine/threonine kinase domain (Torii et al., 1996). *ERECTA* is widely expressed in plant tissues and plays an important role in plant development and responses to external environmental signals (Yokoyama et al., 1998; Shpak, 2013; Jorda et al., 2016; Gao et al., 2017). *ERECTA* knockout mutants show obvious dysplasia phenotypes, for instance, stem node, siliques and petiole become short and inflorescences are densely clustered. Although *Landsberg* deficit in *ERECTA* gene is widely used as an *Arabidopsis* ecotype, there is no in-depth study on how *ERECTA* gene mutations have led to such a markedly intense phenotype. In addition, *ERECTA*, as a multi-functional major gene in the plant, has two homologous genes *ERECTA LIKE 1* (*ERL1*) and *ERECTA LIKE 2* (*ERL2*), both of which have partial functional redundancy with *ERECTA*. The triple mutant *er erl1 erl2* intensifies *ERECTA* knockout mutant phenotype, and the plant is extremely dwarfed (Shpak et al., 2004). Previous studies have been shown that *ERECTA*, *ERL1* and *ERL2* play roles in the organ morphology and size determination in the reproductive stage of anthers and eggs by altering cell cycle progression (Shpak et al., 2004; Pillitteri et al., 2007). The mechanism of *ERECTA* gene family in cell-to-cell communication has been intensively studied in the stomatal development. *ERECTA* can recognize small peptide of the EPF/EPFL family, by forming a complex with TMM, affects stomatal development and differentiation by triggering MAPK cascade (Meng et al., 2012; Jewaria et al., 2013; Lin et al., 2017). However, the role of *ERECTA* family in the regulation hypocotyls remains unclear. Previous studies have shown that overexpression of *YUCCA5*, a member of *YUCCA* family of flavin monooxygenase in *Arabidopsis* auxin synthesis, inhibits mutant phenotype of *er-103* (one of the *ERECTA* mutants) (Woodward et al., 2005). In addition, *ERECTA* family regulates the transport of auxin by regulating the expression of *PIN1* at the initial stage of leaf primordia (Chen et al., 2013). However, the genetic interaction between the *ERECTA* family and auxin signaling in control of the hypocotyl development is still poorly understood.

In this study we found that, the hypocotyl lengths of single mutant *er-105* and triple mutant *er erl1 erl2* were significantly shorter than that of the wild type, whereas hypocotyls of ER-overexpressed plants was longer. We further showed that this change is due to a change in the length of a single cell in the hypocotyl rather than a change in the number of cells. In addition, we found that *DR5::GFP* signal of auxin reporter in the triple mutant *er erl1 erl2* was significantly reduced than that of wild type. Moreover, the transcriptional levels of auxin early response gene and the major auxin synthetase genes were significantly repressed in the *er erl1 erl2* triple mutant, suggesting that the auxin synthesis in the *er erl1 erl2* triple mutant is sabotaged. Consistent with the observation, the exogenous auxin and endogenous auxin were able to rescue the shortened hypocotyl and cell length phenotype of the *er erll erl2* triple mutant, indicating that these defects are due to the lack of auxin synthesis. Therefore we concluded that the *ERECTA* gene family controls the cell elongation in the hypocotyl by positively regulating the auxin biosynthesis in *Arabidopsis thaliana*.

## Materials and Methods

### Plant Materials and Growth Conditions

All of the *Arabidopsis* lines used in this study were in the Col-0 background. The *ER* family mutants and overexpression plants have been described previously (Torii et al., 1996; Shpak et al., 2004; Shen et al., 2015). The seeds for *er-105*, *er-/- erl1+/- erl2-/-* mutant were kindly provided by Prof. Masao Tasaka (Nara Institute of Science and Technology). The seeds for *35S::ERECTA* were kindly provided by Prof. Zuhua He (Shanghai Institutes for Biological Sciences, Chinese Academy of Sciences). The seeds for *DR5::GFP-NLS* in wild type were kindly provided by Prof. Yuling Jiao (Institute of Genetics and Developmental Biology, Chinese Academy of Sciences). Other *Arabidopsis* seeds were obtained from self-preserved, genetic hybridization or agrobacterium-mediated transformation. All seeds were sterilized with 70% ethanol and 0.5% Tween for 10 min, followed by washing two times with 95% ethanol and air drying. After sterilization, seeds were sown onto 1/2 Murashige and Skoog (MS) medium containing 1% sucrose and 0.8% agar, then incubated at 4°C in darkness for 2 days, finally grown vertically at 21°C under long-day condition (16 h of light and 8 h of darkness).

### Plasmid Construction

For construction of *pER::3 × VENUS-NLS*, a 1.4-kb upstream sequence of *ERECTA* before ATG was used as a promoter. For the *pERL1::3 × VENUS-NLS*, a 4.1-kb upstream sequence of *ERL1* before ATG was used as a promoter. For the *pERL2::3 × VENUS-NLS*, a 3.6-kb upstream sequence of *ERL2* before ATG was used as a promoter. For construction of *pER::ER-GFP*, *pERL1::ERL1-GFP* and *pERL2::ERL2-GFP*, the genomic sequence of these three genes were amplified and used the same promoter we described above. The *pER::ER-GFP* construct was then transformed into the *er-105* mutant to rescue its defects, while other constructs were transformed into the Col-0. For p*ER::iaaM*, the 1.4-kb promoter of *ERECTA* was used to drive iaaM sequences that were obtained from Prof. Yunde Zhao (UC San Diego). This construct was then transformed into the *er-/- erl1+/- erl2-/-* mutant. The primer sequences used in the plasmid construction are listed in Supplementary Table S1.

### Gene Expression Analysis

Whole seedlings were dissected and immediately transferred to liquid nitrogen. The Tripure Isolation Reagent (Roche) was used to isolate total RNA from the plant samples. The PrimeScriptTM RT Reagent Kit (TaKaRa) was used for cDNA synthesis. Quantitative PCR was performed with the Thermo PIKO REAL96 Real-Time PCR system using the GoTaq^®^ qPCR Master Mix (Promega) with the following PCR conditions: 95°C for 5 min and 40 cycles of 95°C for 10 s, 57°C for 30 s and 72°C for 30 s, followed by 72°C for 10 min and 20°C for 10 s. *TUBULIN* was used to normalize the mRNA levels. Primers used for qRT-PCR are listed in Supplementary Table S1.

### Propidium Iodide Staining

Propidium Iodide (PI) powder (Sigma) was dissolved with PBS solution to 5 mg/ml as a stock solution, which was stored in dark. The working solution was diluted to 5 μg/ml. The whole seedling was put into the solution for staining 10 min, then washed with water three times and then observed under the confocal microscope (OLYMPUS LSM1200).

### Measurement

For the length of hypocotyls in the seedling stages, pictures were taken under the microscope; The Image J software was used for the measurement and statistical analysis. The cells in longitudinal direction from the top to the base of the hypocotyls epidermis were used for the measurement of each cell length, and 25 hypocotyls were used for biological repetitions, and the Image J software was used for the statistical analysis. The total cells number in the hypocotyls was counted directly under the microscope.

### Exogenous Chemical Substance Treatment

IAA and yucasin powder (Sigma) were dissolved in the DMSO and diluted to 50 μM for IAA and 5 mM for yucasin as the stock solutions. The stock solutions were diluted 1000x and added to the corresponding 1/2MS media. The control group was added with the same amount of DMSO in the media. The plants were grown vertically at 21°C under long-day condition (16 h of light and 8 h of darkness).

### Statistical Analysis

Where appropriate, statistical analyses were performed with analysis of variance (ANOVA) test. Otherwise, comparisons between two groups were conducted using Student’s *t*-test. The *p*-value level was set to 5%.

## Results

### The Hypocotyl Length Is Shortened in the *ERECTA* Family Mutants

One of the most striking defects in the *ERECTA* family mutants (*erf*) mutants is the dwarf phenotype (Shpak et al., 2004). The *er-105* single mutant showed reduced plant height comparing to that of wild type, whereas the plant height was increased in the *ERECTA* overexpressing plants (Supplementary Figure S1A) (Shen et al., 2015). In the *er erl1 erl2* triple mutant, we observed even severe phenotypes in the reduction of the plant height (Supplementary Figure S1A), suggesting that *ERECTA*, *ERL1* and *ERL2* were functionally redundant in control of the plant height. To further support these observations, we measured the plant height of the *35S::ERECTA*, *er-105* and *er erl1 erl2* plants. We observed a slight increase of the plant height in the *35S::ERECTA* plants, however, a significant decrease in the *er-105* and *er erl1 erl2* mutants (Supplementary Figure S1B).

**FIGURE 1:**
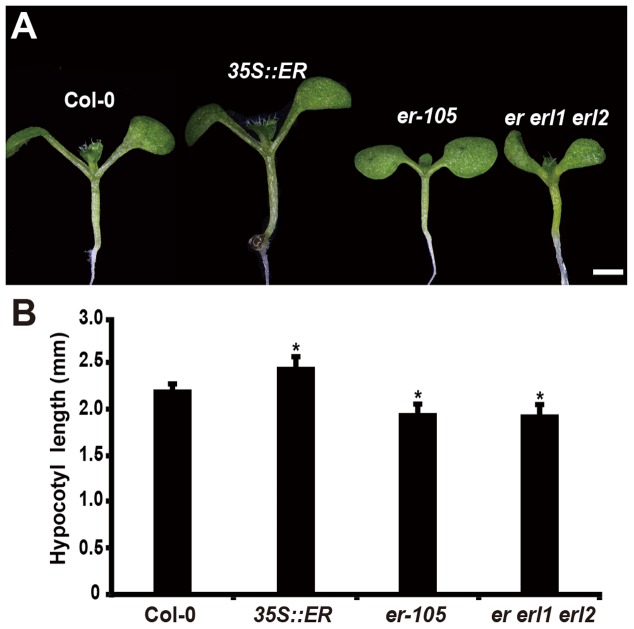
Hypocotyl length is shorten in the *erf* mutants. **(A)** Six-day-old seedlings of the wild-type plant, *35S::ERECTA* transgenic plant, *er-105* mutant and *er erl1 erl2* mutant. **(B)** Average hypocotyl lengths of the 6-day-old seedlings in **(A)** (*n* = 40). Scale bar, 1 mm. ^∗^*P* < 0.05, Student’s *t*-test.

Consistent with the observation of plant height, in the 6-day-old seedling, we observed that the length of hypocotyl in the *35S::ERECTA* plants was significantly increased comparing to that of wild type seedlings (**Figures 1A,B**). Conversely, the hypocotyl lengths in the *er-105* and *er er1 erl2* mutants were dramatically reduced (**Figures 1A,B**). We conclude that the *ERECTA* gene family are involved in the regulation of hypocotyl length in *Arabidopsis*.

### Expression Patterns of *ERECTA* Gene Family in the Hypocotyl

**FIGURE 2:**
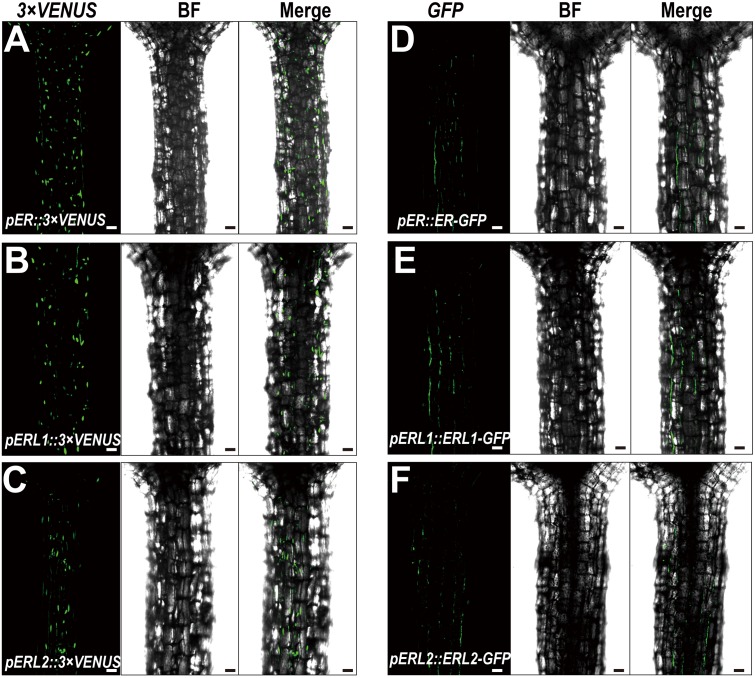
Expression patterns of the *ERECTA* family genes in hypocotyls. **(A)** The expression of the *ERECTA* promoter in hypocotyls determined by the fluorescent signals of *pER::3 × VENNS-NLS*. **(B)** The expression of the *ERL1* promoter in hypocotyls determined by the fluorescent signals of *pERL1::3 × VENNS-NLS*. **(C)** The expression of the *ERL2* promoter in hypocotyls determined by the fluorescent signals of *pERL2::3 × VENNS-NLS*. **(D)** The localization of ERECTA proteins in hypocotyls. **(E)** The localization of ERL1 proteins in hypocotyls. **(F)** The localization of ERL2 proteins in hypocotyls. Scale bars, 50 μm.

Given the fact that disturbing the functions of *ERECTA* gene family causes the severe defects in the hypocotyl length, we assumed that *ERECTA* gene family are expressed in the hypocotyl cells. To test whether the *ERECTA* family genes are transcribed in the hypocotyl cells, we first performed the promoter activation analysis. To this end, we constructed *pER::3 × VENUS-NLS, pERL1::3 × VENUS-NLS, pERL2::3 × VENUS-NLS* transgenic plants. We observed evenly distributed fluorescence signals in the whole hypocotyl of these three transgenic plants (**Figures 2A–C**), suggesting that *ERECTA, ERL1* and *ERL2* genes are expressed in the whole hypocotyl of *Arabidopsis*.

Because *ERECTA* family genes encode leucine-rich repeat receptor-like kinases, we then test whether the ERECTA, ERL1 and ERL2 proteins are localized in the hypocotyl cells. We transformed the *pER::ER-GFP* construct into the *er-105* mutant and observed a fully rescue of the mutant defects. By the confocal microscopy, we observed that ERECTA proteins were strongly accumulated in the hypocotyls (**Figure 2D**).

To check the protein localization of two homologous genes of the *ERECTA*, the *pERL1::ERL1-GFP, pERL2::ERL2-GFP* transgenic plants were also analyzed under the confocal microscope. Consistent with the observation of ERECTA proteins, we found that the both of proteins, ERL1 and ERL2, were also localized in hypocotyls (**Figures 2E,F**).

### The *ERECTA* Gene Family Controls the Cell Elongation of Hypocotyls

**FIGURE 3:**
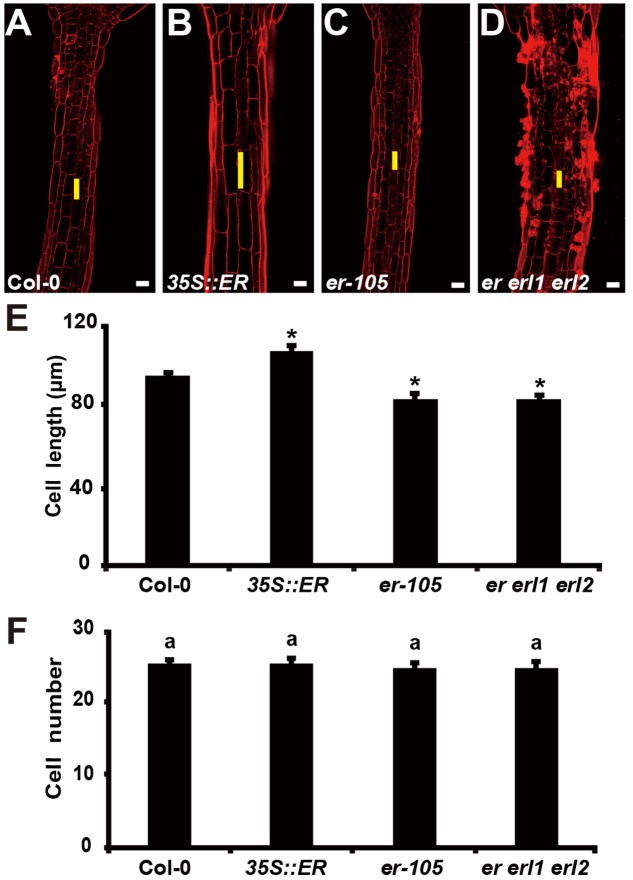
The *ERECTA* family genes controls cell elongation in the hypocotyl. **(A–D)** PI staining of hypocotyls in the 6-day-old wild-type **(A)**, *35S::ERECTA* plant **(B)**, *er-105* mutant **(C)** and *er erl1 erl2* mutant **(D)**. **(E)** Average individual cell lengths of the hypocotyls in the 6-day-old wild-type, *35S::ERECTA* plant, *er-105* mutant and *er erl1 erl2* mutant (*n* = 25). **(F)** Total cell number in the hypocotyls of 6-day-old wild-type, *35S::ERECTA* plant, *er-105* mutant and *er erl1 erl2* mutant (*n* = 25). The yellow line represents the length of a single cell. Scale bars, 50 μm. ANOVA-test in **(F)**, same letters represent no statistically significant differences (*P* < 0.05). Student’s *t*-test in **(E)**, ^∗^*P* < 0.05.

The organ size and shape of multicellular organism is directly related to the cells division and the size of cells. Therefore, the individual cell length and the total number of cells could determine the hypocotyl length in *Arabidopsis*. Previous studies have shown that *ERECTA* family genes may regulate cell number in some organs, including stomata, ovule and stamen (Shpak et al., 2004; Woodward et al., 2005; Pillitteri et al., 2007; Hord et al., 2008). However, whether the *ERECTA* family genes control the cells division and cell elongation in the hypocotyl is still unclear. To test this, we performed Propidium Iodide (PI) staining on the hypocotyls of the wild-type plants, *35S::ERECTA* plants, *er-105* single mutant plants, and *er erl1 erl2* triple mutant plants (**Figures 3A–D**). By the observation under the confocal microscope, we found that the individual cell size of hypocotyl in the *35S::ERECTA* overexpression plants was large than that of wild type plants (**Figure 3B**). On the contrary, we observed size largely reduced cells in the hypocotyls of the *er-105* single mutant and *er erl1 erl2* triple mutant plants comparing to that of wild type plants (**Figures 3C,D**). By the quantitative analysis of the average cells length in the middle column of the hypocotyl epidermis, we found that the average cells length in the *35S::ERECTA* overexpression plants was significantly increased, whereas it was remarkably decreased in the *er-105* and *er erl1 erl2* mutants (**Figure 3E**).

To test whether cells division is involved in the *ERECTA* family genes controlled hypocotyl elongation, we directly counted the total number of cells in the middle column of epidermis, and found that there were no significant difference in all the genotypes we test including the wild type, *35S::ERECTA*, *er-105* and *er erl1 erl2* plants (**Figure 3F**). We concluded that the *ERECTA* gene family regulates the hypocotyl length by controlling the cell elongation rather than the cell division.

### Auxin Biosynthesis Is Down Regulated in the *erf* Mutants

**FIGURE 4:**
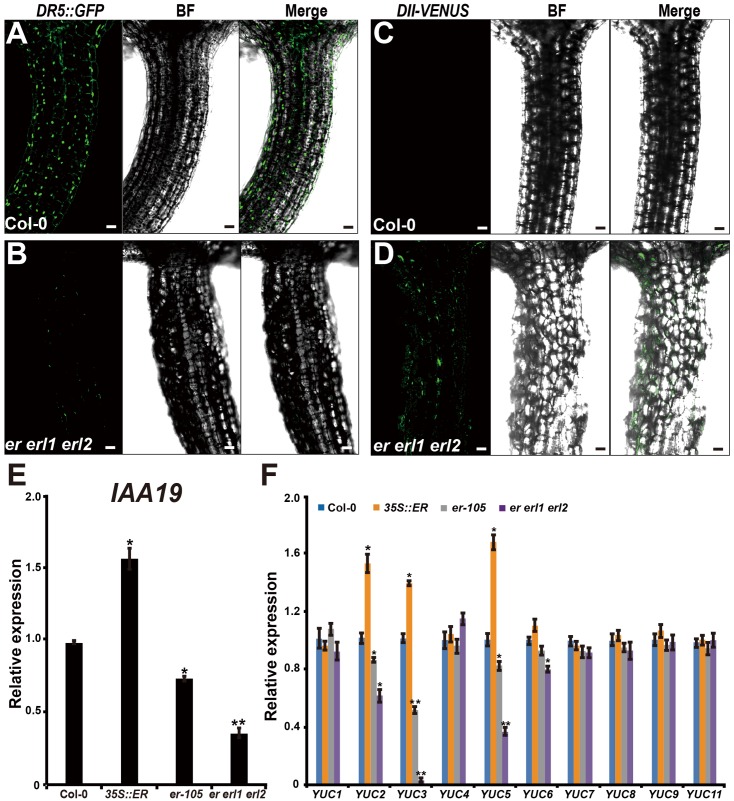
The *ERECTA* family genes controls auxin biosynthesis in the hypocotyl. **(A)** The fluorescent signals of *DR5::GFP* in the wild-type hypocotyl. **(B)** The fluorescent signals of *DR5::GFP* in the *er erl1 erl2* mutant hypocotyl. **(C)** The fluorescent signals of *DII-VENUS* in the wild-type hypocotyl. **(D)** The fluorescent signals of *DII-VENUS* in the *er erl1 erl2* mutant hypocotyl. **(E)** The expression levels of *IAA19* in the wild-type, *35S::ERECTA*, *er-105* and *er erl1 erl2* hypocotyls. The 6-day-old hypocotyls of each genotype were used for the RNA extraction. **(F)** The expression levels of *YUC* family genes in the wild-type, *35S::ERECTA*, *er-105* and *er erl1 erl2* hypocotyls. The 6-day-old hypocotyls of each genotype were used for the RNA extraction. Scale bars, 50 μm. Error bars in the **(E,F)** indicate the SD of three biological repeats. ^∗^*P* < 0.05, ^∗∗^*P* < 0.01, Student’s *t*-test.

Given the fact that the cell elongation was significantly reduced in the *erf* mutants, we then asked by what means does the *ERECTA* gene family regulate individual cell length in the hypocotyl? Previous studies have shown that the auxin plays an important role in regulation of cell elongation (Rayle and Cleland, 1992; Galinha et al., 2007). The endogenous auxin increased mutants exhibit hypocotyls-elongated phenotypes, whereas auxin deficient mutants have the reduced size of cells (Romano et al., 1991; Zhao et al., 2001; Cheng et al., 2006). To examine whether the auxin is involved in the *ERECTA* gene family mediated controls of cell elongation, two different auxin sensors, *DR5::GFP* and *DII-VENUS*, were applied to detect the endogenous auxin levels in the *er erl1 erl2* mutants. The *DR5::GFP* is a positive auxin sensor by which *GFP* transcriptions are activated by auxin (Ulmasov et al., 1997; Benkova et al., 2003). Under the confocal microscope, we observed evenly distributed fluorescence signals of *DR5::GFP* in the entire hypocotyl (**Figure 4A**), suggesting that the auxin was distributed in all the hypocotyl of *Arabidopsis*. However, we can barely detect the fluorescence signals in the *er erl1 erl2* mutant hypocotyl (**Figure 4B**), suggesting that the endogenous auxin levels were dramatically reduced in the triple mutant hypocotyl. To confirm this observation, the transgenic plants that harbored the negative auxin sensor *DII-VENUS* by which fluorescence proteins were degradated by auxin (Brunoud et al., 2012) were further analyzed. Consistent with the observation of *DR5::GFP*, there were only extremely weak signals can be detected in the wild type hypocotyl (**Figure 4C**), but stronger *DII-VENUS* signals in the *er erl1 erl2* triple mutants (**Figure 4D**). At the molecular level, we tested the transcriptional levels of auxin early response gene *IAA19*, which is commonly used to monitor the endogenous levels of auxin in *Arabidopsis*. We observed that the expression levels of *IAA19* in the *35S::ERECTA* transgenic plants were up regulated, whereas significantly down regulated in the *er-105* and *er erl1 erl2* triple mutants (**Figure 4E**). These data suggest that the *ERECTA* gene family is essential to maintain the endogenous auxin levels in the hypocotyl.

To shed light on the molecular mechanism underlying the ability of the *ERECTA* gene family controlling auxin levels in the hypocotyl, we examined the expression levels of the *YUCCA* family genes, which encodes the major auxin biosynthesis genes (Cheng et al., 2006). We observed that the transcriptional levels of *YUC2, YUC3* and *YUC5* were significantly increased in the *ERECTA* overexpression plants compared with wild type (**Figure 4F**). Conversely, these three key auxin biosynthesis genes were markedly decreased in the *er-105* and *er erl1 erl2* triple mutants; especially the expression levels of *YUC3* were drastically reduced in the *er erl1 erl2* triple mutants (**Figure 4F**). We draw the conclusion that the *ERECTA* gene family positively regulates auxin biosynthesis via activating the expressions of *YUC2, YUC3* and *YUC5* genes.

### Exogenous Auxin Rescues the Cells Elongation Defects in *erf* Mutants

**FIGURE 5:**
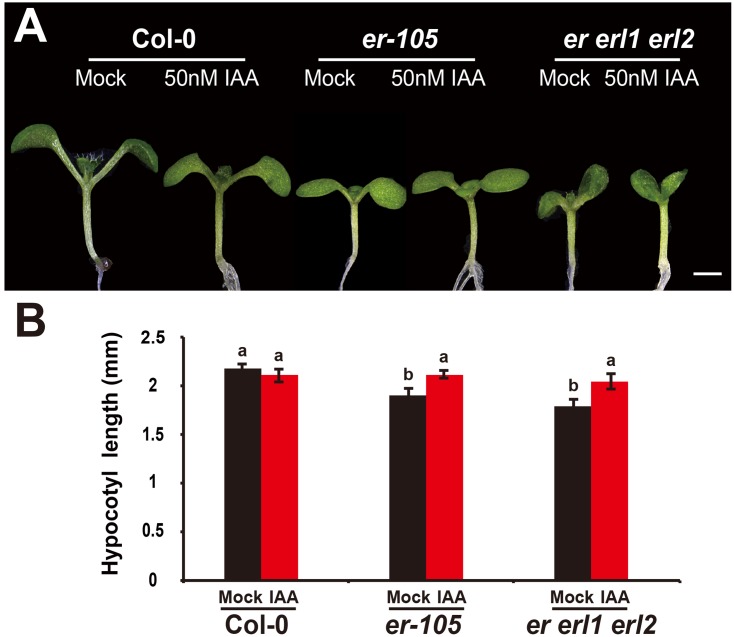
The low auxin rescues the *erf* hypocotyl-shortened phenotypes. **(A)** Six-day-old seedlings of the wild type, *er-105* and *er erl1 erl2* mutant grown in the 1/2MS media with or without 50 nM IAA. **(B)** The hypocotyl lengths of the seedlings in **(A)** (*n* = 40). Scale bar, 1 mm. Different letters represent statistically significant differences (*P* < 0.05), ANOVA-test.

**FIGURE 6:**
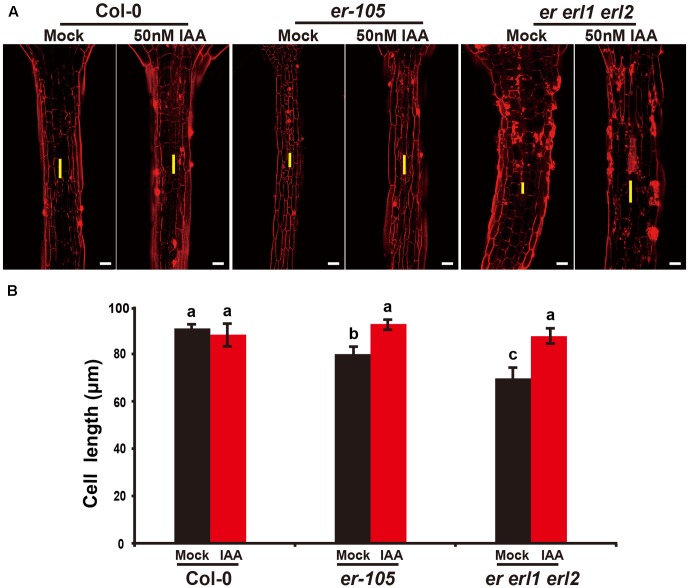
Exogenous auxin rescues the cell elongation defects in the *erf* mutants. **(A)** PI staining of hypocotyls in the wild type, *er-105* and *er erl1 erl2* mutant grown in the 1/2MS media with or without 50 nM IAA. **(B)** The average cell lengths of the hypocotyls in the wild type, *er-105* and *er erl1 erl2* mutant grown in the 1/2MS media with or without 50 nM IAA. (*n* = 25). The yellow line represents the length of a single cell. Scale bars, 50 μm. Different letters represent statistically significant differences (*P* < 0.05), ANOVA-test.

Given the fact that the *erf* mutants contain a very low amount of auxin and the key auxin biosynthesis genes are under the positive control of *ERECTA* gene family, we hypothesized exogenous increase of auxin might rescue the defects of cell elongation and short hypocotyl phenotypes of the *erf* mutants. While, previous studies have shown that the high concentrations of auxin might inhibit the elongation of hypocotyls in wild-type *Arabidopsi*s (Collett et al., 2000; Rashotte et al., 2003), which was also found in our experiments (data not shown). To exclude the possibility the high auxin inhibits the cell elongation in our test, we carefully select a very low concentration of IAA (50 nM), and performed our analysis. By applying this low concentration of IAA to the wild type seedlings, we did not observe any significant differences in term of the hypocotyl length comparing with the mock treatments (**Figures 5A,B**). However, this low concentration of IAA almost fully rescued the hypocotyl-shortened phenotypes of the *er-105* single mutants and *er erl1 erl2* triple mutants (**Figure 5A**). By quantifying the hypocotyl length, we observed significant increases of the hypocotyl length in the *er-105* and *er erl1 erl2* mutants upon the auxin treatments, which showed no differences to that of wild type seedlings (**Figure 5B**), suggesting that the hypocotyl-shortened defects in the *erf* mutants are due to the lacking of auxin, and the increase of exogenous auxin completely restores the mutant phenotypes. To further confirm this observation, we examine the cell elongation in the *erf* mutants after the auxin treatments. In the wild type hypocotyl, the 50 nM auxin treatments did not change the cell length comparing with the mock treatments (**Figures 6A,B**). However, in the *er-105* and *er erl1 erl2* mutants, the same amount of auxin effectively promoted the cell length to that of mock treatments (**Figure 6A**), and the cell length was significantly increased to the levels that comparable with the wild type seedlings (**Figure 6B**). To test the possibility whether the cell division was also involved in the rescue of *erf* mutants defects during the low auxin treatments, we directly counted the total number of cells in the middle column of epidermis with or without the low auxin treatments. We observed no significant difference among all the genotypes after the auxin treatments (Supplementary Figure S2). Therefore, we conclude that the hypocotyl-shortened phenotypes in the *erf* mutants are due to the cell elongation defects, which can be fully rescued by the treatments of exogenous auxin.

### The *ERECTA* Gene Family Mediated Endogenous Auxin Biosynthesis Controls the Cell Elongation in Hypocotyls

Given the fact that the *ERECTA* gene family positively regulates auxin biosynthesis in the hypocotyl by activating the expressions of several *YUCCA* family genes, and the overexpression of *ERECTA* causes the elevated expressions of *YUC*s (**Figure 4F**), we then tested this interaction genetically. We treated the *35S::ERECTA* seedlings with the chemical yucasin, which have been shown to reduce the exogenous auxin levels by inhibiting the expression of *YUCCA* genes (Nishimura et al., 2014). We carefully select a low concentration of yucasin (5 μM) to perform the analysis, which showed no effect on the hypocotyl length in the wild type seedlings (Supplementary Figures S3A,B). While in the *35S::ERECTA* seedlings, the hypocotyl length was significantly decreased (Supplementary Figures S3A,B). Likely, the hypocotyl cell length of the wild type plants did not respond the low yucasin treatments. However, the cell length of the *35S::ERECTA* seedlings with increased *YUCCA* expressions, reduced dramatically upon the low yucasin treatments (Supplementary Figure S4), demonstrating that the *ERECTA* gene family controls the cell elongation in hypocotyls by positively regulating the auxin biosynthesis.

**FIGURE 7:**
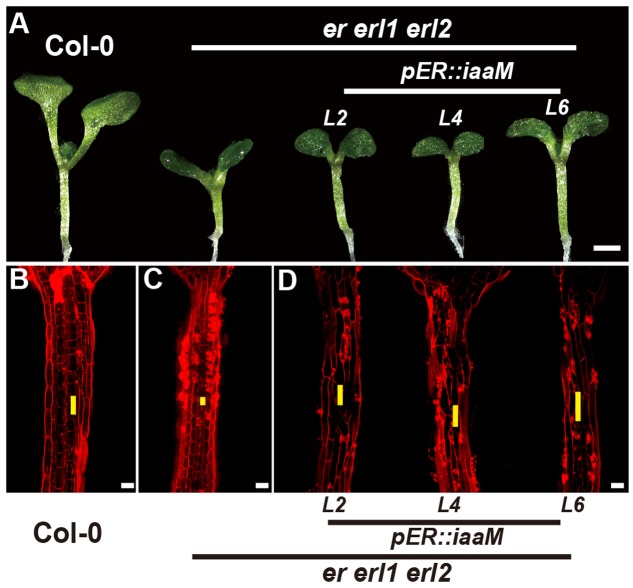
The endogenous auxin rescues the *erf* mutant phenotypes in the hypocotyl. **(A)** Six-day-old seedlings of the wild type, the *er erl1 erl2* mutant and three independent *pER:iaaM* transgenic lines in the *er erl1 erl2* mutant background (*L2, L4 and L6*). **(B–D)** PI staining of hypocotyls in the wild type **(B)**, the *er erl1 erl2* mutant **(C)** and three independent *pER:iaaM* transgenic lines in the *er erl1 erl2* mutant background **(D)**. The yellow line represents the length of a single cell. Scale bar in **(A)**, 1 mm; scale bars in **(B–D)**, 50 μm.

The bacterial auxin biosynthesis gene *iaaM*, which was firstly identified in the T-DNA of agrobacterium, is commonly used in plants to increase the endogenous auxin levels (Comai and Kosuge, 1982). To further investigate the mechanism by which the *ERECTA* gene family mediated auxin synthesis controls the cell elongation in hypocotyls, we expressed the *iaaM* gene under the control of the *ERECTA* promoter, and transformed the *pER:iaaM* construct into the *er-/- erl1+/- erl2-/-* mutants. By analyzing the expression levels of the *iaaM* and the *IAA19* genes in eight independent transgenic lines, we observed a correlation between these two genes, suggesting that the ectopically expressed *iaaM* increased the endogenous auxin levels in the *er erl1 erl2* triple mutant background (Supplementary Figure S5A). We measured the hypocotyl length of eight independent transgenic lines and selected three independent T1 transgenic lines with high expression of *iaaM* for the further analysis. (Supplementary Figure S5B), and observed that hypocotyls and individual cell lengths of *pER:iaaM* transgenic plants were higher than those of *er erl1 erl2* triple mutants (**Figures 7A–D**). Thus we conclude that the *ERECTA* gene family mediated endogenous auxin biosynthesis controls the cell elongation in hypocotyls.

## Discussion

The *ERECTA* family genes, which encode leucine-rich repeat receptor-like kinases, have been shown to regulate multiple developmental processes including stomatal formation, inflorescence architecture, and ovule development (Shpak et al., 2004, 2005; Lee et al., 2012). Woodward et al have previously shown that a dominant mutant, *super1-D*, which causes the overexpression of one of the *YUCCA* family genes *YUC5*, is epistatic to *er* mutants (Woodward et al., 2005). However, little is known about the genetic interaction between the *ERECTA* and auxin signaling in control of the hypocotyl development. In this study, we analyzed the endogenous auxin levels in the *erf* mutants by using two different auxin sensors, *DR5::GFP* and *DII-VENUS*. Our results show that the endogenous auxin levels were dramatically reduced in the hypocotyl of the *er erl1 erl2* triple mutant (**Figures 2A–D**). To further support this observation, we examined the expression levels of *IAA19* in the hypocotyl. We found that the *IAA19* transcripts are significantly reduced in the *er-105* and *er erl1 erl2* mutants, and increased in the *35S::ERECTA* transgenic plants (**Figure 4E**). To further investigate the mechanism by which the *ERECTA* family genes regulate auxin levels in the hypocotyl, we examined the key auxin biosynthesis genes. We observed that the transcriptional levels of *YUC2, YUC3* and *YUC5* were significantly decreased in the *er-105* and *er erl1 erl2* triple mutants, whereas increased in the *35S::ERECTA* transgenic plants (**Figure 4F**). Our data suggest that the *ERECTA* gene family positively regulates auxin biosynthesis by activating the key auxin biosynthesis genes.

Previous study has shown that the overexpression of *YUC5* in the wild type, *er-103* and *er-105* cause the same extent of increase of hypocotyls regardless of their genotypes (Woodward et al., 2005), which raises the question whether the *ERECTA* family genes regulate the cell elongation in the hypocotyl via auxin biosynthesis. By careful selection of a very low concentration, 50 nM of IAA fully rescued the short hypocotyl and cell elongation defects in the *er-105* and *er erl1 erl2* mutants, but have no significant effects in the wild type (**Figures 5, 6**), suggesting that these defects of the *erf* mutants are caused by the auxin deficiency in the hypocotyl. Further support for this idea came from the observation that the elongated hypocotyl cells in the *35S::ERECTA* seedlings are completely suppressed by using the 5 μM yucasin treatments to inhibit *YUCCA* genes (Supplementary Figure S4). Our data demonstrate that the *ERECTA* gene family controls cell elongation by positively regulating auxin biosynthesis. Likely, during leaf margin morphogenesis, the ligand-receptor pair of EPFL2-ER has been shown to be essential for maintaining an appropriate auxin concentration (Tameshige et al., 2016).

The small-secreted peptide PSY1, together with its LRR receptor, positively regulates cell elongation in hypocotyls by triggering a signaling cascade (Mahmood et al., 2014), suggesting a crucial role of peptide ligand-receptor pair in the hypocotyl regulation. ERECTA with its ligands EPF/EPFL peptides family, participate multiple functions during plant development, for example, EPF1, EPF2 and EPFL9 in stomatal regulation, EPFL4, EPFL5 and EPFL6 in inflorescence development and EPFL2 in tooth development (Abrash et al., 2011; Uchida and Tasaka, 2013; Tameshige et al., 2016; Lin et al., 2017). Moreover, in the secondary growth of *Arabidopsis* hypocotyls, *ERECTA* and *ERL1* are very important in the xylem and phloem to prevent premature during sequential events in the secondary growth, suggesting that *ERECTA* family plays an important role in the morphogenesis of hypocotyls (Ikematsu et al., 2017). Therefore, we speculate that ERECTA family might regulate the cell elongation in hypocotyls with some members of the EPF/EPFL family or other ligands.

During the reproductive stage, the short internodes and pediceels phenotypes of the *erf* mutants in the inflorescences were mainly due to reduced cell proliferation (Shpak et al., 2003, 2004; Woodward et al., 2005). However, in the hypocotyl, mutations of the *ERECTA* gene family result in the defects specifically in the cell elongation rather than the cell division (**Figure 3**). This idea is further support by the early observation that, in *Arabidopsis*, hypocotyl elongation is mainly due to the cell elongation, while the number of cells is rather stable (Gendreau et al., 1997). These data suggest that the functions of *ERECTA* gene family in controlling of cell proliferation and cell elongation is highly tissue-specific, however, it remains to be shown how the functions of *ERECTA* gene family are specified in different tissues to fulfill their biological functions.

## Author Contributions

ZT, XQ, and ZZ designed the experiments, analyzed the data and wrote the paper. XQ performed the experiments.

## Conflict of Interest Statement

The authors declare that the research was conducted in the absence of any commercial or financial relationships that could be construed as a potential conflict of interest.
