# Supplementary material for: ERECTA Regulates Cell Elongation by Activating Auxin Biosynthesis in Arabidopsis thaliana
Source: Front Plant Sci. 2017 Sep 27;8:1688. doi: 10.3389/fpls.2017.01688 (PMC5623719; doi:10.3389/fpls.2017.01688)
Supplement: Supplementary file 1 [file Data_Sheet_1.docx]

Supplementary Material

***ERECTA*** **regulates** **cell elongation by activating auxin biosynthesis in *Arabidopsis thaliana***

**Xiaoya Qu^1^,** **Zhong Zhao^1^* and Zhaoxia Tian^1^***

***Correspondence:**

Zhong Zhao: zhzhao@ustc.edu.cn

Zhaoxia Tian: zxtian@ustc.edu.cn

# Supplementary Figures and Tables

## Supplementary Figures


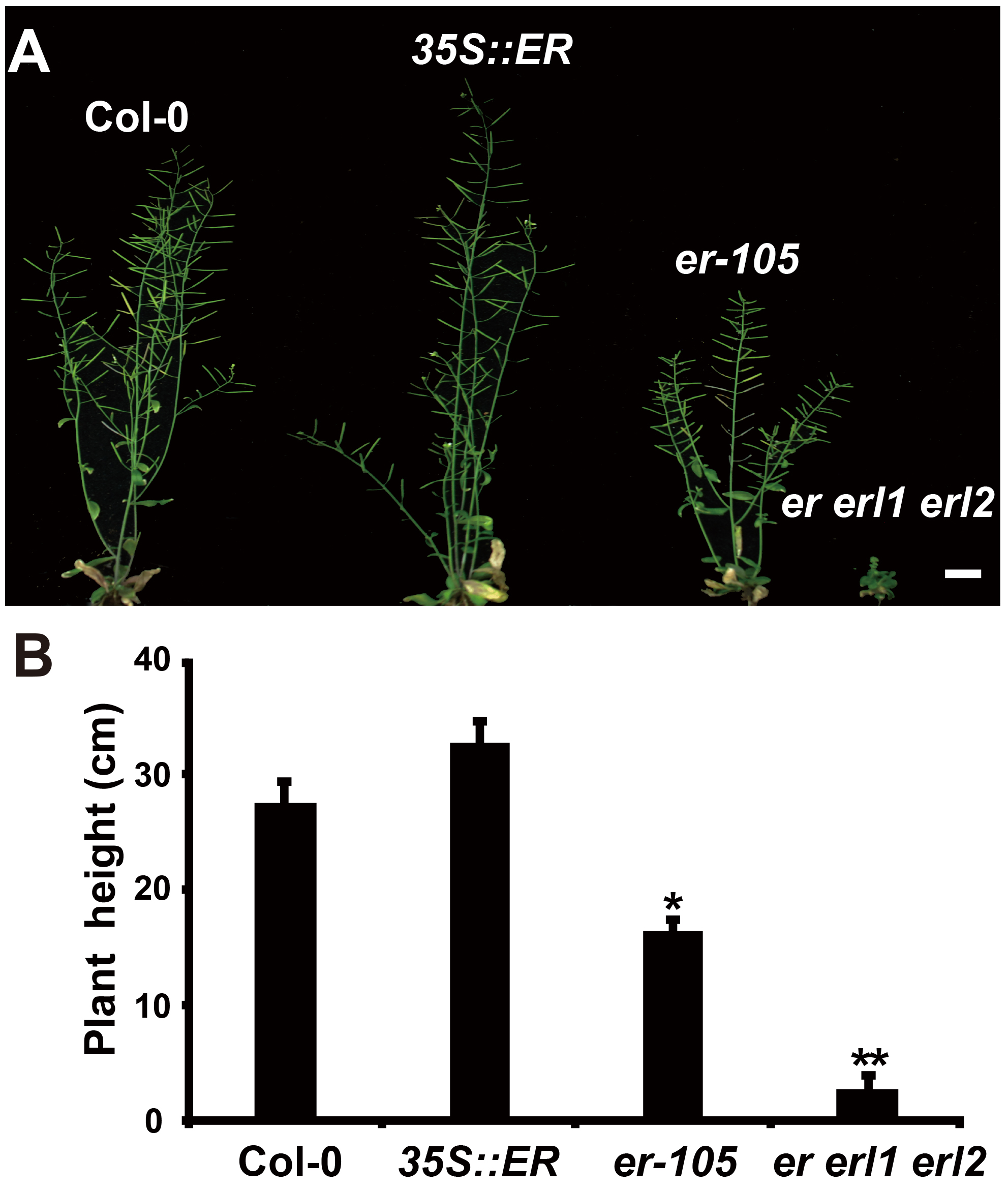


**Supplementary Figure 1.** **Phenotypes of the *erf* mutants**

(A) The 45-day-old of wild-type, *35S::ERECTA,* *er-105* and *er erl1 erl2* plants*.* (B) The plant height of the 45-day-old of wild-type, *35S::ERECTA,* *er-105* and *er erl1 erl2* plants (n=15). Scale bar, 2cm. *P < 0.05, **P < 0.01, Student’s t-test.

**
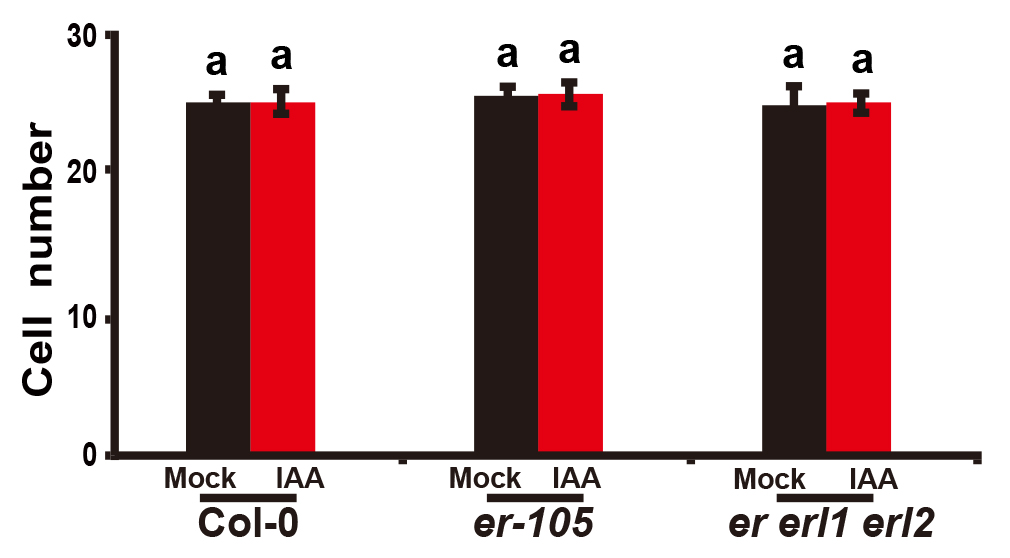
**

**Supplementary Figure 2. The low exogenous auxin did not affect the cell number of hypocotyls**

The total cell numbers in the hypocotyls of the wild type, *er-105* and *er erl1 erl2* mutants were count with or without 50 nM IAA treatment. Same letters represent no statistically significant differences (p > 0.05), ANOVA-test.

**
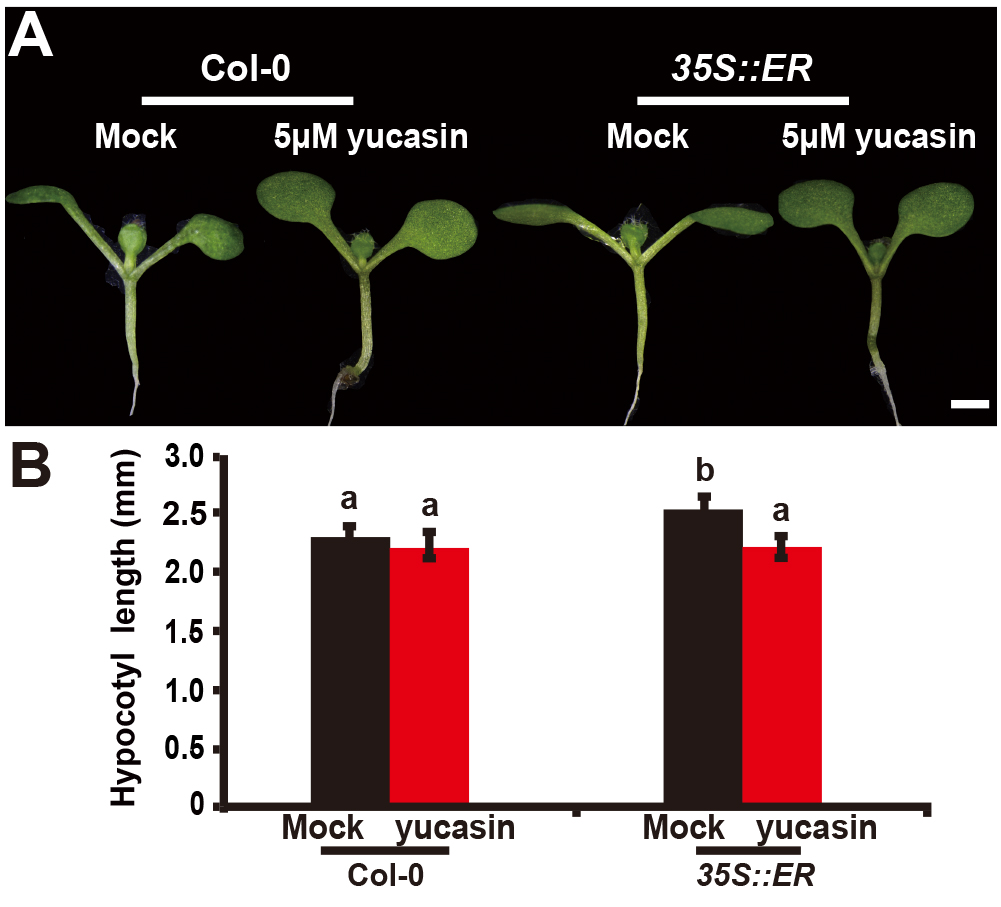
**

**Supplementary Figure 3. The yucasin treatments reverse the elongated hypocotyl phenotypes in the *35S::ERECTA* plants**

(A) Six-day-old seedlings of the wild type and *35S::ERECTA* plants grown in the 1/2MS media with or without 5 μM yucasin. (B) The hypocotyl lengths of the seedlings in (A) (n=40). Scale bar, 1mm. Different letters represent statistically significant differences (p < 0.05), ANOVA-test.

**
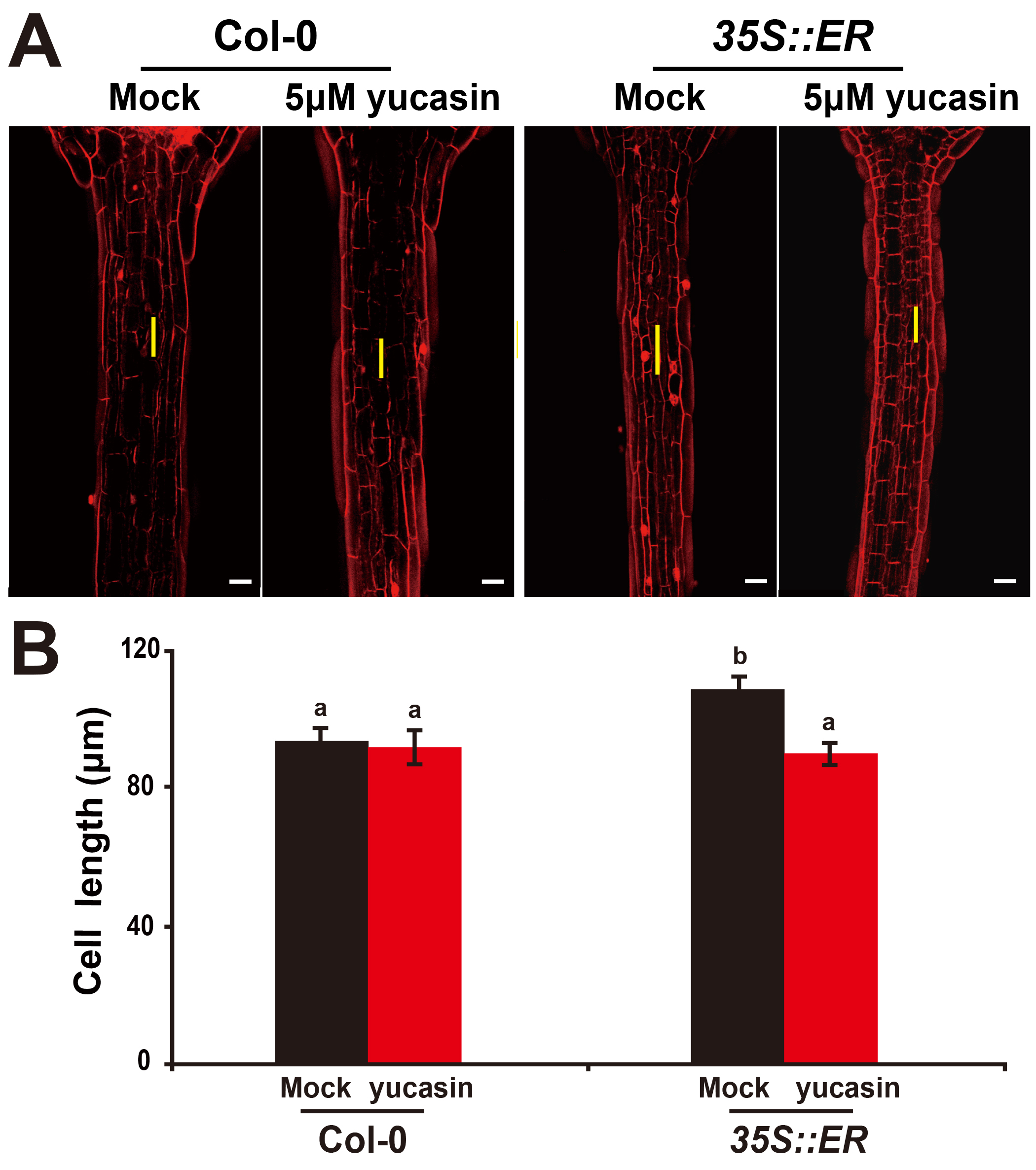
**

**Supplementary Figure 4.** **The yucasin treatments repress the cell elongation in the hypocotyls of *35S::ERECTA* plants**

(A) PI staining of hypocotyls of the wild type and *35S::ERECTA* plants grown in the 1/2MS media with or without 5 μM yucasin. (B) The average cell lengths of the hypocotyls in the wild type and *35S::ERECTA* plants grown in the 1/2MS media with or without 5 μM yucasin. The yellow line represents the length of a single cell. Scale bars, 50 μm. Different letters represent statistically significant differences (p < 0.05), ANOVA-test.

**
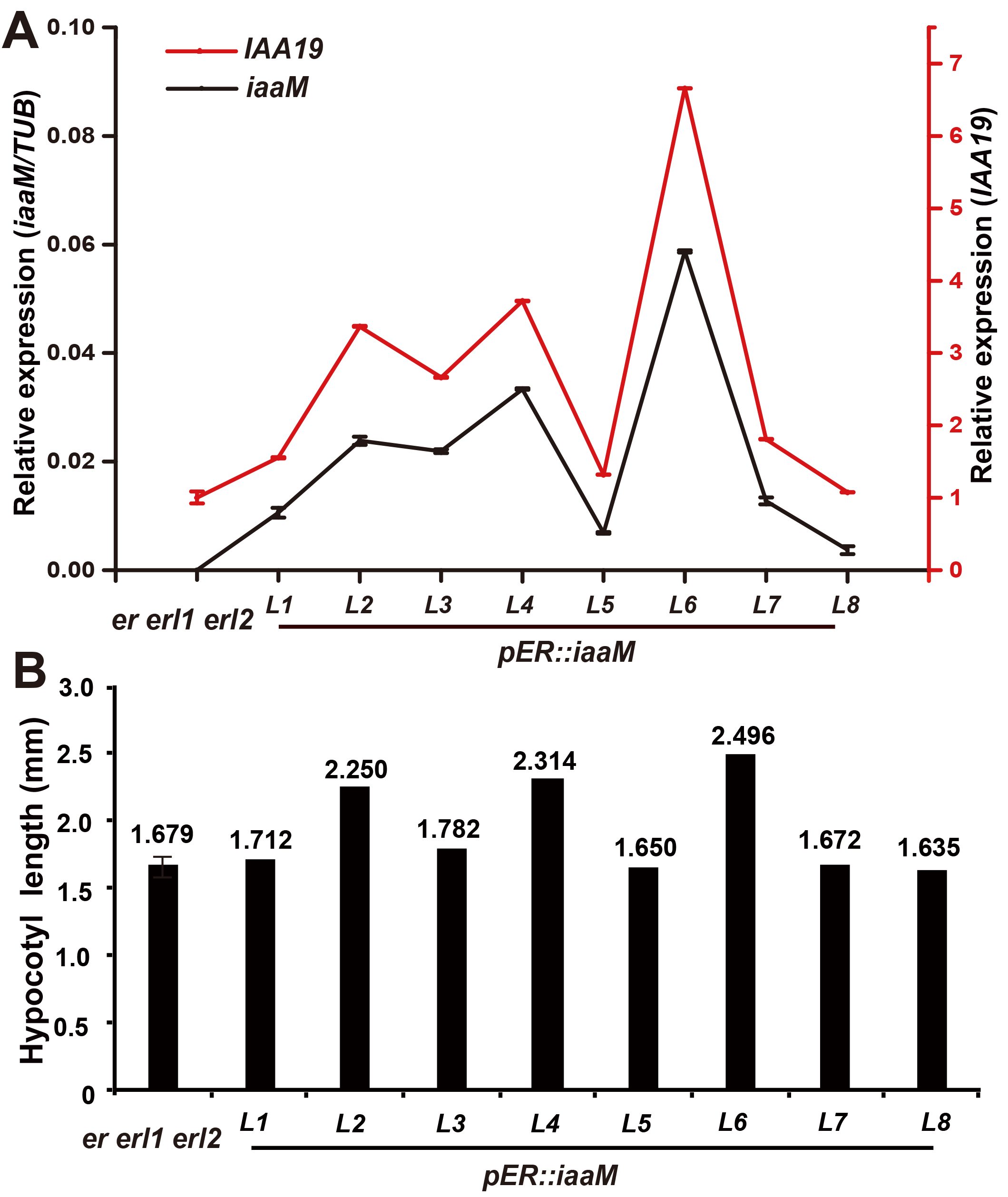
**

**Supplementary Figure 5. The *pER: iaaM* increases the endogenous auxin levels in the *er erl1 erl2* mutants.**

(A)The quantitative analysis of the exogenous *iaaM* and the endogenous *IAA19* expression levels in 8 independent *pER: iaaM* transgenic lines in the *er erl1 erl2* mutant background at the seedlings stages.

(B) Hypocotyl lengths of the 6-day-old seedlings of 8 independent *pER: iaaM* transgenic lines in the *er erl1 erl2* mutant background.

## Supplementary Tables

**Supplemental Table 1: Primer sequences used this study**

| **Purpose** | **Gene** | **Name** | **Sequence (from 5’ to 3’)** |
| --- | --- | --- | --- |
| qRT-PCR | *TUBULIN* | H0069 | GAGCCTTACAACGCTACTCTGTCTGTC |
|  |  | H0070 | ACACCAGACATAGTAGCAGAAATCAAG |
|  | *IAA19* | H0073 | TGGTGTGCCTTATTTGAGGAAG |
|  |  | H0074 | CCGGTAGCATCCGATCTTTT |
|  | *YUC1* | H0694 | CGGTCGGATTCAATAGCATCTC |
|  |  | H0695 | AAGCGTAGGACTCAAGGTAGG |
|  | *YUC2* | H0696 | GGATGAGACAATGGAGTATG |
|  |  | H0697 | ATATTTCACCGCTCTTATAGG |
|  | *YUC3* | H6235 | ATCAACCCTAAGTTCAACGAGACA |
|  |  | H6236 | CGGCACAACTTTCTCAGCG |
|  | *YUC4* | H0698 | ACGCATCTGGTCTATGGAATG |
|  |  | H0699 | CGGACTTGTACGCACTGG |
|  | *YUC5* | H6028 | TCGTCCCGGGAATTAAACGGTTCT |
|  |  | H6029 | CCGATTTCCCTTTCCACGCGTTT |
|  | *YUC6* | H0700 | GGTTGAGTCGGCTGCGTTTG |
|  |  | H0701 | ACATACTCCGTCGTGCCTTCTTC |
|  | *YUC7* | H2937 | GTGGAAAGGGAAGGCTGGATTG |
|  |  | H2938 | ACAACGACGGTGGCGAGTA |
|  | *YUC8* | H6030 | CAAGGAGTCCCATTCGTTGTGGT |
|  |  | H6031 | TCGAACCGGTTTGCGTATGACTCA |
|  | *YUC9* | H6237 | ATCACTACCCTGAATACCCAACG |
|  |  | H6238 | TTCTAACCCTCCATAGCCCG |
|  | *YUC10* | H6239 | TTCGCCACCAAACTCTTTACC |
|  |  | H6240 | GTCTTCCCGTTGATGCTTCC |
|  | *YUC11* | H6241 | ACACTCCTACCTTCGTCTCC |
|  |  | H6242 | CGCAGCAACCATAAACTTCG |
|  | *iaaM* | H5525 | ACGCTCCTAGTGTCGTGGC |
|  |  | H5526 | TCCACATGTATTGGACGCC |
| Plasmid construction | *ERECTA* promoter | H2690 | AATTTAATTAGAAATTTGCGGATG |
|  |  | H2691 | AGAGGATAGAGAAGAAGAAGAGAAC |
|  | *ERL1* promoter | H1860 | ACACCAATAAAAATACACAGCA |
|  |  | H1861 | TTCTTCTTATTCTTCTTTCCTTTTGG |
|  | *ERL2* promoter | H1862 | GTGATTAGGAGACGAGGTAGATA |
|  |  | H1863 | CTTCTTCTTCTTCTTCCTCAAGA |
|  | *ERECTA genomic* | H2168 | ATGGCTCTGTTTAGAGATATTG |
|  |  | H2172 | TACTCACTGTTCTGAGAAATAAC |
|  | *ERL1 genomic* | H2173 | ATGAAGGAGAAGATGCAGCG |
|  |  | H2177 | TATATGCTACTTTTGGAGATGAC |
|  | *ERL2*  *genomic* | H2178 | ATGAGAAGGATAGAGACCATG |
|  |  | H2182 | TATAAGCTACTTTTGGAGATATC |
